# Utilities and Limitations of the World Health Organization 2009 Warning Signs for Adult Dengue Severity

**DOI:** 10.1371/journal.pntd.0002023

**Published:** 2013-01-17

**Authors:** Tun-Linn Thein, Victor C. Gan, David C. Lye, Chee-Fu Yung, Yee-Sin Leo

**Affiliations:** 1 Communicable Disease Centre, Tan Tock Seng Hospital, Singapore, Singapore; 2 National University of Singapore, Yong Loo Lin School of Medicine, Singapore, Singapore; Pediatric Dengue Vaccine Initiative, United States of America

## Abstract

**Background:**

In 2009, the World Health Organization (WHO) proposed seven warning signs (WS) as criteria for hospitalization and predictors of severe dengue (SD). We assessed their performance for predicting dengue hemorrhagic fever (DHF) and SD in adult dengue.

**Method:**

DHF, WS and SD were defined according to the WHO 1997 and 2009 dengue guidelines. We analyzed the prevalence, sensitivity (Sn), specificity (Sp), positive predictive value (PPV) and negative predictive value (NPV) of WS before DHF and SD onset.

**Results:**

Of 1507 cases, median age was 35 years (5^th^–95^th^ percentile, 17–60), illness duration on admission 4 days (5^th^–95^th^ percentile, 2–6) and length of hospitalization 5 days (5^th^–95^th^ percentile, 3–7). DHF occurred in 298 (19.5%) and SD in 248 (16.5%). Of these, WS occurred before DHF in 124 and SD in 65 at median of two days before DHF or SD. Three commonest warning signs were lethargy, abdominal pain/tenderness and mucosal bleeding. No single WS alone or combined had Sn >64% in predicting severe disease. Specificity was >90% for both DHF and SD with persistent vomiting, hepatomegaly, hematocrit rise and rapid platelet drop, clinical fluid accumulation, and any 3 or 4 WS. Any one of seven WS had 96% Sn but only 18% Sp for SD.

**Conclusions:**

No WS was highly sensitive in predicting subsequent DHF or SD in our confirmed adult dengue cohort. Persistent vomiting, hepatomegaly, hematocrit rise and rapid platelet drop, and clinical fluid accumulation, as well as any 3 or 4 WS were highly specific for DHF or SD.

## Author Summary

Dengue is a mosquito-borne infection with significant public health burden in tropical and subtropical regions. Clinical presentations may vary from self-limiting fever to severe dengue including death. The World Health Organization 2009 dengue guidelines classified dengue into dengue with and without warning signs, and severe dengue. In our adult dengue cohort, we found that lethargy, abdominal pain/tenderness, and mucosal bleeding occurred commonly. As predictors of severe dengue, these were not as specific as persistent vomiting, hepatomegaly, hematocrit rise and rapid platelet drop, and clinical fluid accumulation which occurred infrequently. Presence of any one warning sign had high sensitivity, but low specificity for severe dengue. Removal of lethargy from the list increased the specificity of any one warning sign, but sensitivity decreased. Consideration of these findings may avoid over hospitalization of potentially non-severe dengue patients and reduce burden on the healthcare system.

## Introduction

Dengue is an acute febrile disease with a wide range of clinical presentations. The disease has been known for more than a century in the tropical areas of South East Asia and the Western Pacific regions [1]. Epidemics of dengue have now become a regular occurrence worldwide [2]. Although various diagnostic tests are available for diagnosis of dengue [3], [4], predicting disease outcome of dengue patients remains challenging. Early identification of signs that can predict severe dengue (SD) may save lives by facilitating early initiation of interventions and frequent monitoring [5].

Attempts at providing evidence-based predictors for severe disease have been made. A study on adult dengue inpatients in Singapore showed that dengue hemorrhagic fever (DHF) can be predicted by the presence of bleeding, hypoproteinemia, raised blood urea and lymphopenia [6]. Ramirez-Zepeda et al reported the presence of ascites, gum bleeding, hematemesis, thrombocytopenia and persistent vomiting as predictors of major complications [7]. Thomas et al demonstrated that abdominal pain, cough or diarrhea reported by patients at presentation were significantly associated with the development of severe manifestations [8]. A study from India reported that profound shock was preceded most commonly by sudden hypotension [9].

The World Health Organization (WHO) and the Special Program for Research and Training in Tropical Diseases (TDR) jointly published the dengue guidelines for diagnosis, treatment, prevention and control in 2009, suggesting seven warning signs to identify patients at risk of SD. The proposed warning signs include abdominal pain or tenderness, persistent vomiting, clinical fluid accumulation, mucosal bleed, lethargy or restlessness, liver enlargement >2 cm, increase in hematocrit concurrent with rapid decrease in platelet count [10]. Barniol et al reported that the use of these warning signs for case management was of practical value [11]. Alexander et al described these warning signs associated with disease progression in a majority of younger patients [12]. In Singapore, 92.2% of dengue cases reported to Ministry of Health were older than 14 years [13]. Therefore, these warning signs need to be validated in adult dengue.

We aimed to validate the utility of warning signs from the WHO 2009 guideline in laboratory-confirmed adult dengue inpatients. In particular, we aimed to determine the predictive value of warning signs to predict DHF as defined by the WHO 1997 guideline as well as SD as defined by the WHO 2009 guideline [10], [14].

## Methods

### Ethics statement

Domain Specific Review Board, National Healthcare Group, Singapore approved the study. All data were anonymized.

### Patients

All patients were managed using a standardized dengue clinical care path that facilitated consistent capture of clinical and laboratory data. This retrospective study was performed by extracting demographic, clinical, laboratory, radiological, treatment and outcome data from laboratory-confirmed dengue inpatients managed by the Department of Infectious Diseases at Tan Tock Seng Hospital, Singapore in the years 2004, 2007 and 2008. The study cohorts were selected to represent different predominant dengue serotypes, being dengue serotype 1 (DEN1) in 2004 and dengue serotype 2 (DEN2) in 2007 and 2008 [13]. Our center is the largest single center in Singapore managing predominantly adult dengue, with nearly 40% of nationally reported cases in 2005 [15]. We included only patients with positive dengue polymerase chain reaction (PCR) during the early febrile viremic phase of their illness [3].

### Warning signs

Warning signs recorded include: abdominal pain or tenderness, persistent vomiting, clinical fluid accumulation (pleural effusion or ascites detected by physical examination or radiologically), mucosal bleed, lethargy, hepatomegaly, rise in hematocrit concurrent with rapid drop in platelet count [10]. Lethargy was not included in analysis of 2004 cohort as the information was not available. Clinical outcomes were DHF as defined by the WHO 1997 guideline, and SD as defined by the WHO 2009 guideline. Patients were diagnosed as DHF if they had fever, thrombocytopenia, bleeding and evidence of plasma leakage (either hypoproteinemia, change in hematocrit of more than 20%, or clinical fluid accumulation) [14]. Severe dengue was fulfilled if there was evidence of plasma leakage associated with shock or respiratory distress, severe bleeding or severe organ involvement [10], [14].

### Clinical outcomes

Clinical outcomes were determined using data from the time of hospital presentation to the time of hospital discharge. The exact day patients fulfilled criteria for DHF and SD was noted. Warning signs were analyzed until patients reached the clinical outcomes, i.e., developed DHF and/or SD. Cases with warning signs only occurring after clinical outcomes had reached were excluded from analysis.

### Data analysis

For descriptive analyses, frequency and percentages were used for categorical variables. For continuous variables, median, range and percentiles were used. Warning signs preceding development of clinical outcomes were classified as true positive (TP) cases. Likewise, presences of warning signs in patients without DHF and/or SD were classified as false positive (FP) cases. Sensitivity (Sn), specificity (Sp), positive predictive value (PPV) and negative predictive value (NPV) were determined for individual warning sign as well as for presence of any warning signs in predicting the clinical outcomes of DHF and/or SD. Predictive values for the presence of more than one WS were explored. The Statistical Package for the Social Sciences version 16 (SPSS Inc., Chicago, IL) was used for data analyses.

## Results

### Cohort description

There were 1,507 patients with PCR-confirmed dengue in our study: 917 in 2004, 318 in 2007 and 272 in 2008. Overall, the median age was 35 years (5^th^–95^th^ percentile, 17.0–59.7 years), and 1,037 (68.8%) were male. Majority was Chinese which constituted 1,110 (73.7%). In terms of clinical outcomes, 294 (19.5%) patients developed DHF and 248 (16.5%) patients had SD. One hundred and fifteen patients fulfilled both DHF and SD criteria. Among 248 patients with SD, 56.9% had severe plasma leakage with shock or respiratory distress, 37.9% had severe bleeding and 16.1% had severe organ involvement. Demographic and pertinent clinical data for each year were detailed in Table 1.

**Table 1:** Demographic and clinical characteristics, treatment and clinical outcomes of adult dengue patients with positive dengue polymerase chain reaction.

	Year 2004 (n = 917)	Year 2007 (n = 318)	Year 2008 (n = 272)	Total (n = 1507)
Median age (5^th^–95^th^ percentile)	32 (17–57)	35 (20–62.3)	35 (20–62)	35 (17–59.7)
Male	619 (67.5)	225 (70.8)	193 (71)	1037 (68.8)
Ethnicity				
Chinese	698 (76.1)	219 (68.9)	193 (71)	1110 (73.7)
Indian	79 (8.6)	35 (11)	30 (11)	144 (9.6)
Malay	52 (5.7)	14 (4.4)	17 (6.3)	83 (5.5)
Others	88 (9.6)	50 (15.7)	32 (11.8)	170 (11.3)
Singaporean	564 (61.5)	140 (44)	127 (46.7)	831 (55.1)
Dengue hemorrhagic fever	56 (6.1)	137 (43.1)	101 (37.1)	294 (19.5)
Severe dengue	92 (10)	87 (27.4)	69 (25.4)	248 (16.5)
Median days of illness at presentation (5th–95th percentile)	4 (2–7)	4 (1–6)	4 (2–6)	4 (2–6)
Median days hospitalized (5th–95th percentile)	5 (3–7.3)	5 (3–8)	5 (3–7)	5 (3–7.8)
Intravenous fluid	635 (69.2)	303 (95.3)	242 (89)	1180 (78.3)
Platelet transfusion	124 (13.5)	33 (10.4)	25 (9.2)	182 (12.1)
Admission to intensive care unit	4 (0.4)	2 (0.6)	0 (0)	6 (0.4)

Variables shown are numbers with percentage in parentheses unless otherwise stated.

### Warning signs

Table 2 showed the occurrence of warning signs during the entire clinical course (i.e. from hospital presentation to hospital discharge) as well as up to the day of clinical outcomes of DHF or SD. In our study, 787/1507 (52%) patients had any of six warning signs (without lethargy) during clinical course, of which 125/294 (43%) occurred before progression to DHF and 62/248 (25%) before progression to SD. The three most common warning signs occurring before development of DHF and/or SD were lethargy, abdominal pain or tenderness, and mucosal bleeding. Detailed frequencies of warning signs are shown in Table 2.

**Table 2:** Prevalence of warning signs (WS) during the entire clinical course, and before the development of dengue hemorrhagic fever (DHF) and severe dengue (SD).

		DHF (n = 294)		SD (n = 248)	
Warning signs	Entire clinical course (n = 1507)	Entire clinical course	Before DHF	Entire clinical course	Before SD
**Individual WS**					
Abdominal pain or tenderness	462 (31)	134 (45.6)	66 (22)	109 (44)	36 (15)
Persistent vomiting	130 (9)	42 (14.3)	17 (6)	41 (16.5)	19 (8)
Hepatomegaly	76 (5)	10 (3.4)	3 (1)	7 (2.8)	1 (0)
Hematocrit rise and rapid platelet count drop	351 (23)	74 (25.2)	21 (7)	107 (43.1)	7 (3)
Clinical fluid accumulation	22 (1)	49 (16.7)	6 (2)	55 (22.2)	4 (2)
Mucosal bleeding	177 (12)	208 (70.7)	63 (21)	124 (50)	25 (10)
Lethargy^x^	270 (46)	113 (38.4)	61 (26)	78 (31.5)	40 (26)
**WS count**					
Any number of seven WS^x^	509 (86)	219 (74.5)	124 (52)	153 (61.7)	65 (42)
Any number of six WS (without lethargy)	787 (52)	264 (89.8)	125 (43)	223 (89.9)	62 (25)
One WS	456 (30)	89 (30.3)	82 (28)	72 (29)	53 (21)
Two WS	215 (14)	79 (26.9)	46 (16)	66 (26.6)	21 (8)
Three WS	112 (7)	66 (22.4)	17 (6)	52 (21)	8 (3)
Four WS	43 (3)	24 (8.2)	3 (1)	22 (8.9)	2 (1)
Five WS	15 (1)	14 (4.8)	0 (0)	12 (4.8)	1 (0.4)
Six WS	2 (0.1)	2 (0.7)	0 (0)	1 (0.4)	0 (0)
Seven WS	1 (0.07)	1 (0.3)	0 (0)	1 (0.4)	0 (0)

x Years 2007 and 2008 cohorts only, Variables shown are numbers with percentage in parentheses.

### Analysis for DHF

Table 3 describes the performance of warning signs to predict DHF in 1507 patients. Having any of seven warning signs had 87% Sn to predict DHF, while having any of six warning signs (without lethargy) was marginally lower at 81%. Specificity was high for persistent vomiting (93%), hepatomegaly (99%), rise in hematocrit concurrent with rapid platelet count drop (92%) and clinical fluid accumulation (98%). However, Sp was low at 18% for any of seven warning signs, which improved to 57% if lethargy was omitted. Positive predictive values of warning signs were low, varying from 16% to 31%. Only any of six warning signs without lethargy and mucosal bleeding had NPV exceeding 90%. Performances for the presence of strictly one or more than one WS in predicting DHF were also shown. The occurrence of three or four warning signs was associated with specificity of 96% and 98% respectively. The performance for warning signs for each year was found to be similar to the overall cohort analysis although there was a difference in predominant serotype (data not shown).

**Table 3:** Performance of warning signs (WS) for predicting dengue hemorrhagic fever (DHF) (n = 1507).

Warning signs	Sn	Sp	PPV	NPV
**Individual WS**				
Abdominal pain or tenderness	0.29	0.73	0.17	0.85
Persistent vomiting	0.06	0.93	0.16	0.82
Hepatomegaly	0.01	0.99	0.20	0.81
Hematocrit rise and rapid platelet count drop	0.09	0.92	0.17	0.83
Clinical fluid accumulation	0.02	0.98	0.18	0.83
Mucosal bleeding	0.42	0.88	0.31	0.93
Lethargy^x^	0.33	0.55	0.28	0.61
**WS count** *				
Any number of seven WS^x^	0.87	0.18	0.30	0.77
Any number of six WS (without lethargy)	0.81	0.57	0.19	0.96
One WS	0.64	0.70	0.18	0.95
Two WS	0.44	0.89	0.25	0.95
Three WS	0.21	0.96	0.27	0.95
Four WS	0.04	0.98	0.14	0.94

Sn = sensitivity, Sp = specificity, PPV = positive predictive value, NPV = negative predictive value,

x Years 2007 and 2008 cohorts only,

* Median number of WS before DHF was 1 (range, 1–4).

### Analysis for SD

Table 4 showed the performance of warning signs to predict SD for all patients. Having any of seven warning signs had 96% Sn to predict SD, while having any of six warning signs (without lethargy) had 71% Sn to predict SD. Persistent vomiting (93%), hepatomegaly (99%), rise in hematocrit concurrent with rapid platelet count drop (94%), and clinical fluid accumulation (98%) had good Sp for SD. Specificity was higher without lethargy (55% for any of six warning signs without lethargy versus 18% for any of seven warning signs). Positive predictive value for SD was even lower than for DHF, ranging from 6% to 18%. Individual warning signs had negative predictive value for SD less than 90%, compared with 96% for any one of seven warning signs and 97% for any one of six warning signs (without lethargy). The occurrence of three or four warning signs was associated with specificity of 95% and 98% respectively.

**Table 4:** Performance of warning signs (WS) for predicting severe dengue (SD) (n = 1507).

Warning signs	Sn	Sp	PPV	NPV
**Individual WS**				
Abdominal pain or tenderness	0.21	0.72	0.09	0.87
Persistent vomiting	0.08	0.93	0.18	0.85
Hepatomegaly	0.00	0.99	0.06	0.84
Hematocrit rise and rapid platelet count drop	0.05	0.94	0.09	0.89
Clinical fluid accumulation	0.02	0.98	0.16	0.87
Mucosal bleeding	0.17	0.82	0.10	0.89
Lethargy^x^	0.34	0.56	0.17	0.76
**WS count** *				
Any number of seven WS^x^	0.96	0.18	0.15	0.96
Any number f six WS (without lethargy)	0.71	0.55	0.10	0.97
One WS	0.58	0.69	0.12	0.96
Two WS	0.32	0.88	0.12	0.96
Three WS	0.15	0.95	0.12	0.96
Four WS	0.04	0.98	0.25	0.96
Five WS	0.02	1.00	0.09	0.96

Sn = sensitivity, Sp = specificity, PPV = positive predictive value, NPV = negative predictive value,

x Years 2007 and 2008 cohorts only,

* Median number of WS before SD was 1 (range, 1–5).

### Duration of warning signs from illness onset

Most warning signs appeared at median illness day 4 (5^th^–95^th^ percentile, 2–7 days) and preceded DHF or SD at a median of 2 days before progression to DHF (5^th^–9^th^ percentile, 1–4 days) or SD (5^th^–9^th^ percentile, 1–6.1 days) (Table 5). Median duration from onset of individual warning signs to development DHF or SD were statistically different for DHF (p<0.05 by Kruskal-Wallis test), but not for SD (p = 0.80 by Kruskal-Wallis test).

**Table 5:** Duration from the onset of warning signs to dengue hemorrhagic fever (DHF) and severe dengue (SD).

Warning signs	Days to DHF*	Days to SD*
Abdominal pain or tenderness	1 (1–4)	2 (8–2)
Persistent vomiting	2 (1–6)	2 (1–5.7)
Hepatomegaly	3 (3–3.9)	1.5 (1–2)
Hematocrit rise and rapid platelet count drop	1 (1–3)	3 (1–7.6)
Clinical fluid accumulation	2 (1–5)	3 (1–8)
Mucosal bleeding	2 (1–4)	2 (1–8.5)
Lethargy^x^	2 (1–5)	3 (1–8)

* Median (5^th^–95^th^ percentiles),

x Years 2007 and 2008 cohorts only.

### Intensive care unit (ICU) admission and death

Among the six intensive care unit (ICU) admissions, five patients had warning signs; of these four patients presented with WS prior to ICU admission. Of the five, four cases had DHF and all five cases progressed to SD. The sole fatality fulfilled DHF (two days after admission) and SD criteria (on admission day) and had three WS, two of which occurred before DHF, namely abdominal pain or tenderness, and rise in hematocrit concurrent with rapid platelet count drop.

## Discussion

Warning signs were reported by Guzman et al in a study of 12 adult DHF deaths where 58.3% of patients manifested warning signs on the second or third day from illness onset, of which abdominal pain and persistent vomiting were the commonest and most frequent [16]. Of 17 patients with DHF grade IV in Lucknow, India (both children and adults), the commonest warning sign was sudden hypotension (47%); among the warning signs reported that were included in the WHO 2009, vomiting occurred in 23%, severe abdominal pain and restlessness in 18% each [9]. In a study of 23 dengue deaths in Puerto Rico (both children and adults), Rigau-Perez et al noted that any one of clinical alarm signs occurred in 48%, usually occurring on the day of deterioration; of these, severe abdominal pain occurred in 26% and persistent vomiting in 13% [17].

These important observations were subsequently supported by several studies that examined symptoms and signs at presentation to predict dengue severity. Carlos et al in a study of 359 children with dengue of which a third had DHF reported that restlessness, epistaxis and abdominal pain at hospital presentation were significantly associated with DHF (P<0.05) [18]. In a study of 560 adults with dengue at Martinique, France, where severe dengue was defined as hypotension, encephalopathy, plasma leakage, platelet count <20×10∧9/liter, aminotransferase levels more than 10 times upper limit of normal, and severe bleeding, the sensitivity and specificity for predicting these subsequent severe manifestations for symptoms at hospital presentation were: abdominal pain, 62.6% and 52.7%; cough, 37.6% and 77.3%; and diarrhea, 65.9% and 74.4% [8].

In a study of 79 children and adults requiring major intervention versus 691 controls in Southeast Asia and Latin America, Alexander et al found that abdominal pain or tenderness, lethargy, mucosal bleeding and platelet decrease noted one day prior within days 4 to 7 of illness were independently associated with need for major interventions [12]. These data from the DENCO study provided the basis for the WHO 2009 recommendations of warning signs [10]. In a study of 181 children with dengue in Rio de Janeiro, Brazil, where 30 patients had severe dengue according to the WHO 2009 criteria, lethargy and abdominal pain were independently associated with severe dengue, but not vomiting, clinical fluid accumulation, hepatomegaly and bleeding [19].

We systematically studied the utility of warning signs as proposed by the WHO 2009 for predicting DHF and SD as defined by the WHO in 1997 and 2009 respectively. The first observation was that while warning signs occurred in 86% of 1507 adult dengue confirmed with PCR, lower proportion occurred before development of the two clinical outcomes, 52% for DHF and 42% for SD. If lethargy was removed, then the overall incidence decreased from 86% to 52%, for DHF from 52% to 43% and for SD from 42% to 25%. For warning signs that did occur before DHF or SD, the median duration was 1–3 days for DHF and 1.5–3 days for SD, which allowed a window of opportunity for intervention. The second observation was that many warning signs on their own were uncommon, but lethargy, abdominal pain or tenderness and mucosal bleeding were the three commonest occurring before the development of DHF or SD. Persistent vomiting, hepatomegaly, rise in hematocrit concurrent with rapid drop in platelet count, and clinical fluid accumulation occurred in less than 10%. The third observation was that the removal of lethargy in our adult cohort improved the specificity of any of remaining six warning signs for DHF or SD. The fourth observation was that four warning signs were highly specific for both DHF or SD, namely persistent vomiting, hepatomegaly, rise in hematocrit concurrent with rapid drop in platelet count, and clinical fluid accumulation, albeit they occurred infrequently.

Our analysis in a large cohort of PCR-positive adult dengue patients from three years with predominant dengue serotypes 1 and 2 showed that the positive predictive value for DHF and SD were not high, ranging from 16% to 31% for DHF, and 6% to 18% for SD. This finding was compatible with findings by Kalayanarooj in a study of 247 children with dengue and 24 without dengue that warning signs occurred in 50% of non-dengue and 53.3% of dengue fever [20]. There may be variations among children and adults with dengue as warning signs occurred in 52% of DHF and 42% of SD in our study of adult dengue versus 83–100% in DHF grades I to IV of pediatric dengue [20]. As warning signs are recommended as criteria of hospitalization [10], both sensitivity and specificity are important. High specificity is important to optimize the use of scarce hospital resources to manage patients at high risk of progressing to severe dengue [21]. At the same time sensitivity is critical to ensure that dengue patients are not being sent home with subsequent progression to DHF or SD. Our analysis showed that individual warning signs failed to fulfill this requirement for adult dengue. However, while any one of seven warning signs was associated with 95% sensitivity and 96% negative predictive value, its specificity of 18% may result in over-hospitalization if this were to be used as a criterion for hospital admission.

Limitations of our study included its retrospective study design, study population limited to adult dengue, study setting in a developed country with all cases confirmed with PCR and hospitalized in a tertiary referral center, lack of primary or secondary dengue infection data, dengue serotype data in individual patients, and lack of data on lethargy in our 2004 cohort. Despite its retrospective nature, all patients were managed with a standardized dengue clinical care path which helped to improve the reliability of data on vital signs, full blood count, serum creatinine and aminotransferases, and clinical data vital to the classification of warning signs, DHF and SD. Our large sample size of 1507 PCR-positive adult dengue with 294 DHF and 248 SD contributed to better understanding of warning signs for dengue severity where available data were mainly in children. Notably, we did not study the utility of warning signs as diagnostic criteria for probable dengue [10] as all our patients were confirmed with PCR, and we could not assess their utility as admission criteria [10] as all our patients were hospitalized.

In conclusion, our analysis showed that individual warning signs had low positive predictive value for DHF and SD in a large cohort of PCR-confirmed adult dengue with predominantly dengue serotypes 1 and 2, occurring before the development of DHF in 52% and SD in 42%. Removal of lethargy as a warning sign in adults may improve specificity of remaining six warning signs for both DHF and SD. Common warning signs of lethargy, abdominal pain or tenderness, and mucosal bleeding were not as specific as less frequently observed warning signs of persistent vomiting, hepatomegaly, rise in hematocrit concurrent with rapid drop in platelet count, and clinical fluid accumulation.

## Supporting Information

Checklist S1 **STROBE checklist for the reporting of observational studies.**
(DOC)
